# Comparison of Dynamic Amplification Factor of Deflection and Bending Moment of Highway Continuous Box-Girder Bridges by Mode Superposition

**DOI:** 10.3390/ma17051041

**Published:** 2024-02-24

**Authors:** Yelu Wang, Yongjun Zhou, Yang Zhao, Yu Zhao, Yuxin Xue, Wei Feng

**Affiliations:** 1 School of Highway, Chang’an University, Xi’an 710064, China; 2017021032@chd.edu.cn (Y.W.);; 2 Key Laboratory of Bridge Detection Reinforcement Technology Ministry of Communications, Xi’an 710064, China; 3 Shaanxi Provincial Transport Planning Design and Research Institute Co., Ltd., Xi’an 710065, China; 4 Xi’an Municipal Engineering Design and Research Institute Co., Ltd., Xi’an 710064, China

**Keywords:** bridge engineering, dynamic amplification factor, mode superposition, deflection, bending moment, cut-off mode

## Abstract

There are differences between the dynamic deflection and bending moment (strain) in the same section of continuous girder bridges. However, the selection of the response for calculating dynamic amplification factors (DAFs), which are essential for bridge health monitoring and safety assessment, remains controversial. Modes may play a role in the relationship between the deflection DAF and the bending moment DAF in both numerical analysis and field tests. To investigate the distinctions between the DAFs of the deflection and bending moment in a continuous girder bridge, functional expressions of the DAFs were derived, taking into account multi-factor coupling under concentrated forces. The interaction effects of the mode and road surface condition (RSC), vehicle speed, bridge span length, and span number on the deflection DAF, the bending moment DAF, and the ratio of the deflection DAF to the bending moment DAF (RDM) of precast continuous box-girder bridges were analyzed using vehicle-bridge interaction. To ensure the accuracy of the DAF in numerical computations and experimental tests, two types of accuracy indexes and the corresponding cut-off modes were provided. Validation was conducted by performing dynamic load tests on two field bridges. The results indicate that different modes have a significant effect on the RDM of the mid-span section of a bridge. When considering multiple factors, the deflection DAF and bending moment DAF of the mid-span section increased rapidly with the considered modes and then stabilized. Statistically, the RDM of all nine bridges ranged from 1.00 to 1.12, indicating that the deflection DAF was greater than the bending moment DAF. The suggested cut-off modes can be utilized for efficient and accurate calculation of the DAF and response signal fidelity.

## 1. Introduction

Small- and medium-span bridges are extensively used, with their live load effects accounting for a significant proportion of the bridge effects, making the vehicle-bridge interaction (VBI) effect significant [1,2,3]. The effect of the increased vertical dynamic load produced by vehicles on bridges is usually characterized by a dynamic amplification factor (DAF) [4]. Accurate evaluation of the DAF plays a critical role in the design and evaluation of bridges. A reasonable DAF leads to safer and more economical designs of new bridges and provides valuable information for the condition assessment and management of bridges in service [5,6].

Since the 1950s, significant efforts have been made to study the DAFs on bridges due to vehicle loading. The maximum bending moment and maximum deflection in a static state tend to occur together near the mid-span, so the corresponding DAFs have received much attention. Many researchers have found differences in DAFs obtained from different dynamic responses of a bridge through field tests or numerical simulations. Notably, there have been different views on the relationship between the deflection DAF and the bending moment (strain) DAF obtained at the same position. When the beam is kept in an elastic state, there is a correspondence between the strain and the bending moment. The AASHO road test [7,8] showed that the impact factor (IM, IM = DAF-1) obtained from measured deflection was always higher than that obtained from measured strains. Similar findings were also reported by other researchers. Mohseni [9] formulated the DAFs for bending moment and deflection based on an extensive numerical study involving 125 composite slab-on-girder prototype bridges with simple support. Szurgott [10] conducted experimental tests on a reinforced concrete simply supported girder bridge and found that the DAFs derived from deflection were significantly larger and less trustworthy than those recorded from strains. The deflection was clearly overestimated, including the actual deflection of the girders, the bearing displacements, and the contraction of the bridge piles. Li [11] examined a concrete type-III girder bridge and found that the IMs of deflection were greater than those of the bending moment at mid-span, especially for light trucks. The difference between the two decreased as the weight of the vehicle increased. Huang [12] utilized numerical methods to analyze seven three-span single-cell steel–concrete composite continuous girder bridges, concluding that the IMs of deflection usually exceed those of vertical bending. Majka [13] analyzed the dynamic characteristics of simply supported railway girder bridges through an efficient numerical model and stated that the DAFs of deflection might be conservative compared with those of strain in some cases. Yang [14] analyzed the DAFs of deflection and bending moments for both simply supported beams and continuous beams and showed that the IMs of deflection exceed those of bending moments at the mid-span.

Nonetheless, other researchers reported the opposite conclusion. Aluri [8] demonstrated, using test data from three FRP deck bridges, that the IMs obtained from deflection were lower than those acquired through strain measurements. Fafard [15] researched a cracked continuous concrete skewed girder bridge, and the numerical results showed that the DAF calculated with deflections was smaller than that estimated with strains and reactions. Senthilvasan [16] conducted an experimental study on a continuously curved concrete bridge, showing that strain has a greater dynamic amplification than deflection.

Through careful examination of the above studies, possible reasons for the deviations in the conclusions were identified: (1) whether the bridges are made of the same material or not: the relationship between strain and deflection may be different for steel, concrete, and FRP materials; (2) whether the structure is in an elastic state or not: cracks may change the structural response; (3) different methods of analyzing the structure: the accuracy of measurements made in the field test methods is affected by the performance of sensors and the environment; (4) different structural forms: straight bridges, curved bridges, and straight and skewed bridges may affect the results; (5) whether the measurements are taken at the same point or not: it was observed in some studies in the literature that the deflection and strain measurement points were not located at the edge of the section (upper or lower edge); (6) girder support conditions: girder deflections may include the compression of bearings or piers.

Since the available field test data are limited and tests are costly, numerous theoretical derivations and numerical simulations have been carried out to calculate the bridge responses to vehicle loads [17]. A plethora of scholars have extensively investigated the factors influencing DAFs, including the bridge characteristics, vehicle speed, bridge deck conditions, vehicle loads, etc. [12,17,18,19,20]. Existing studies have also reported the influence of modes on the DAFs owing to the prevalent use of the mode superposition technique [18,21,22]. Through theoretical extrapolation, Humar [23] deduced that the DAFs between the bending moment and deflection of simply supported bridges are equivalent when considering only the first-order modes. However, their precise relationship is contingent upon the contribution of higher-order modes, though they are closely approximated. Li et al. [11] found that all the modes of the bridge are excited when a vehicle is traveling on one side of the bridge, and the first, third, and fifth modes contribute the most to the deflection response, which could be identified from the experiment data. Zhou et al. [24] performed a numerical analysis using a three-axis vehicle model. They found that for simply supported beams, the strain at the bottom of the beam at mid-span is significantly influenced by the higher-order modes, while the deflection is primarily influenced only by the first few modes. Zou et al. [25] showed that the lower-order modes dominate the deflection of the bridge, whereas bending and acceleration are more sensitive to the higher-order modes, necessitating the incorporation of higher-order bridge modes to ensure accurate results for bridge bending moments. Similar findings were also reported by other researchers [22,25,26,27]. In dynamic load tests, the acquired signals are usually filtered and de-noised, resulting in the removal of higher-order signals [16,28]. Currently, there is no consensus on the cut-off modes for calculating the DAFs of continuous girder bridges [29]. The above studies indicated that modes may potentially contribute to the relationship between the deflection DAF and bending moment DAF in numerical analyses and field tests. 

It is, therefore, clear that more research is needed to understand the potential relationship between the deflection DAF, the bending moment DAF, and modes for continuous girder bridges. This will enable their more effective utilization in engineering practice. The primary scope of this study is (1) to clarify the magnitude relationship between the deflection and bending moment of straight precast concrete continuous girder bridges by theoretical and numerical methods; (2) to analyze the effect of vibration modes on the ratio of the deflection DAF to the bending moment DAF, including the coupled effects of some parameters (e.g., vehicle parameters, road surface condition, bridges); and (3) to propose different levels of cut-off modes to reduce the DAF errors caused by modes in numerical analysis or experimental tests. The outline of the paper is as follows: Section 2 presents the theory of the relationship between the DAFs of deflection and bending moments for two-span continuous beams. In Section 3, the development and verification process of the mode superposition method program for VBI analysis is presented. Section 4 analyzes the interactive effects of sensitive parameters and modes on the DAFs of the deflection and bending moment. Section 5 identifies the main vibration modes of the deflection and bending moment and provides the cut-off modes for different computational accuracies. Finally, Section 6 presents an experimental validation, and Section 7 concludes the study.

## 2. Theoretical Analysis

**Figure 1:**
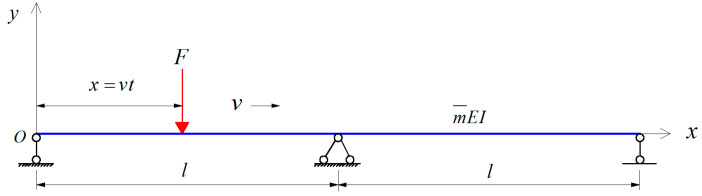
Schematic of a two-span continuous beam.

Take a two-span continuous beam as an example, as shown in Figure 1; assuming that the span is l and the velocity and displacement of the bridge are zero at the initial time, the vibration equation of the bridge subjected to a concentrated force *F* can be expressed as follows:

(1) $$EI\frac{\partial^{4}y_{d}(x,t)}{\partial x^{4}}+\bar{m}\frac{\partial^{2}y_{d}(x,t)}{\partial t^{2}}+c\frac{\partial y_{d}(x,t)}{\partial t}=F(x,t)$$

(2) F(x,t)=Fδ(x−vt)

where v denotes the load speed; t represents time; $y_{d}$ is the vertical displacement of the beam; δ(x−vt) is the Dirac function; F is a moving load; EI is the vertical flexural stiffness of the beam.

According to the principle of mode superposition [30], the deflection and bending moment of the beam can be expressed as follows:

(3) $$y_{d}(x,t)=\sum_{n=1}^{N}\varphi_{n}(x)q_{n}(t)\;n=1,\;2,3..$$

(4) $$M_{d}(x,t)=-EI\frac{\partial^{2}y(x,t)}{\partial x^{2}}=-EI\sum_{n=1}^{N}\frac{\partial^{2}\varphi_{n}(x)}{\partial x^{2}}q_{n}(t)$$

where $\varphi_{n}(x)$ denotes the vibration mode function; $q_{n}(t)$ denotes the generalized modal coordinates; $M_{d}(x,t)$ is the bending moment of the beam at position *x* at time t.

The vibration mode $\varphi_{n}(x)$ of the beam is divided into two cases.

When the mode order is odd, i.e., *n* = 1, 3, 5..., the mode function of the beam can be described as follows:

(5) $$\varphi_{n}(x)=\sin\frac{(n+1)\pi x}{2l}$$

From Equations (1) to (3) and Equation (4), the following equations can be obtained:

(6) $$\omega_{n}=\frac{{\left(n+1\right)}^{2}\pi^{2}}{4}\sqrt{\frac{EI}{\bar{m}l^{4}}}$$

(7) $$q_{n}(t)=\frac{F}{\bar{m}l\omega_{n}^{2}}H_{2}(t)$$

(8) $$\begin{array}{ll} H_{2}(t)= & \frac{1}{{\left(1-\beta_{n}^{2}\right)}^{2}+{\left(2\zeta_{n}\beta_{n}\right)}^{2}}\cdot [e^{-\zeta_{n}\omega_{n}t}\frac{\zeta_{n}\omega_{n}\left(2\zeta_{n}\beta_{n}\right)-\left(1-\beta_{n}^{2}\right)\Omega_{n}}{\omega_{D}}\cdot \sin(\omega_{D}t)+\\ & \left(1-\beta_{n}^{2}\right)\sin(\Omega_{n}t)+e^{-\zeta_{n}\omega_{n}t}\left(2\zeta_{n}\beta_{n}\right)\cos(\omega_{D}t)] \end{array}$$

(9) $$\Omega_{n}=\frac{(n+1)\pi v}{2l}$$

where $\bar{m}$ is the mass of the beam section;$\beta_{n}=\frac{\Omega_{n}}{\omega_{n}}$ is the frequency ratio, which is the ratio of the nth generalized excitation frequency $\Omega_{n}$ to the flexural frequency $\omega_{n}$ of the beam, $\omega_{n}=a_{n}^{2}\sqrt{\frac{EI}{\bar{m}l^{4}}}$; $\omega_{D}=\omega_{n}\sqrt{1-\xi_{n}^{2}}$; $\xi_{n}$ is the damping ratio of the nth-order vibration mode.

When the mode order is even, i.e., *n* = 2, 4, 6..., the mode function of the beam must be given as a piecewise function. 

When 0≤x≤l, the mode function is

(10) $$\varphi_{n}(x)=\sin(\frac{\alpha_{n}x}{l})-\frac{\sin(\alpha_{n})}{\sinh(\alpha_{n})}\cdot \sinh(\frac{\alpha_{n}x}{l})$$

where $\alpha_{n}=3.927+\left(\frac{n}{2}-1\right)\cdot \pi$.

From the combination of Equations (1) to (3) and Equation (9), the following equation can be obtained:

(11) $$q_{n}(t)=\frac{F}{M_{n}\omega_{D}}\cdot \int_{0}^{t}\left\{\left[\sin(\frac{\alpha_{n}vt}{l})-\frac{\sin(\alpha_{n})}{\sinh(\alpha_{n})}\sinh(\frac{\alpha_{n}vt}{l})\right].\right.\left.e^{-\zeta_{n}\omega_{n}(t-\tau )}\sin\omega_{D}(t-\tau )\right\}d\tau$$

where $M_{n}=\int_{0}^{l}\varphi_{n}{\left(x\right)}^{2}\bar{m}\;dx$; τ is the integration factor related to time.

When l≤x≤2l, the mode function is

(12) $$\varphi_{n}(x)=\sin(\alpha_{n}\frac{2l-x}{l})-\frac{\sin(\alpha_{n})}{\sinh(\alpha_{n})}\cdot \sinh(\alpha_{n}\frac{2l-x}{l})$$

From the combination of Equations (1) to (3) and Equation (11), the following equation can be obtained:

(13) $$q_{n}(t)=\frac{F}{M_{n}\omega_{D}}\cdot \int_{\frac{l}{v}}^{t}\left\{\left.\left[\int \sin\left(\alpha_{n}\frac{2l-x}{l}\right)-\frac{\sin(\alpha_{n})}{\sinh(\alpha_{n})}.\right.\sinh\left(\alpha_{n}\frac{2l-x}{l}\right)\right]\cdot e^{-\zeta_{n}\omega_{n}(t-\tau )}\sin\omega_{D}(t-\tau )\right\}d\tau$$

where $M_{n}=\int_{0}^{l}\varphi_{n}{\left(x\right)}^{2}\bar{m}\;dx$; $\alpha_{n}=3.927+\left(\frac{n}{2}-1\right)\cdot \pi$.

The static deflection and bending moment of the beam are

(14) $$\left\{\begin{array}{l} y_{s}(x)=(\lambda_{1}(x)Fl^{3})/EI\\ M_{s}(x)=\lambda_{2}(x)Fl \end{array}\right.$$

where $\lambda_{1}(x)$ and $\lambda_{2}(x)$ are the static load factors of the deflection and bending moment related to the location of the section, respectively. 

When considering the first K order modes of the beam, the DAFs for the deflection and bending moment of the beam can be derived as follows:

(15) $$DAF_{u}=\frac{y_{d}(x,t)}{y_{s}(x)}=g_{y}(\lambda_{1},\frac{x}{l},\frac{v}{l},\xi_{1}\ldots \xi_{K},\beta_{1}\ldots \beta_{K},K)$$

(16) $$DAF_{M}=\frac{M_{d}(x,t)}{M_{s}(x)}=f(\lambda_{2},\frac{x}{l},\frac{v}{l},\xi_{1}\ldots \xi_{K},\beta_{1}\ldots \beta_{K},K)$$

where *N* is the total number of modes of the beam considered.

From Equations (15) and (16), the DAF of the two-span continuous beam is a function of the position coefficient x/l, frequency ratio $\beta_{n}$, static load factor $\lambda_{i}$, damping ratio $\xi_{i}$, and v/l. The damping ratio $\xi_{i}$ of a beam ranges from 0.02 to 0.05 and therefore has a minor effect on the DAF. Once the section position is determined, $\lambda_{i}$ and x/l are constant values. The number of modes considered could affect the accuracy of the DAFs. The more modes are chosen, the more accurate the DAF is.

For two equal-span continuous beams, the ratio of the deflection DAF to the bending moment DAF (RDM) at l/2 is

(17) $$\mathrm{RDM}(\frac{l}{2})=\frac{{\mathrm{DAF}}_{u}}{{\mathrm{DAF}}_{M}}=-\frac{312}{23l^{2}}\times \frac{\sum_{n=1}^{N}\left[q_{n}\left(t\right)\varphi_{n}(x)\right]}{\sum_{n=1}^{N}\left[q_{n}\left(t\right)\frac{\partial^{2}\varphi_{n}(x)}{\partial x^{2}}\right]}$$

The RDM at l/2 is obtained when only the first-order mode is considered as follows:

(18) $$\mathrm{RDM}(\frac{l}{2})=\frac{{\mathrm{DAF}}_{u}}{{\mathrm{DAF}}_{M}}=\frac{312}{23\pi^{2}}\approx 1.37$$

From Equation (18), it can be seen that for two equal-span continuous beams, the deflection DAF is larger than that of the bending moment when only the first-order modes are considered, and the RDM is close to 1.37. When more modes are considered, the results of Equation (15) are dependent on factors such as the span and stiffness, necessitating the use of numerical solution methods. Nevertheless, it is clear that the RDM varies with the number of modes K.

## 3. Vehicle-Bridge Interaction Models

By the previous theoretical derivation, the functional relationship equation between the DAF of the bridge and the related factors subjected to concentrated force is obtained. The bridge modes have a significant effect on the RDM of continuous girder bridges. To further clarify the regularity pattern of DAFs with modes, the influence of real vehicles should be considered. At the same time, possible differences caused by vehicle parameters, the road surface condition (RSC), and bridge types need to be considered.

### 3.1. Vehicle and Bridge Vibration Equations

Vehicle vibration equations can be obtained by the direct balance method and Lagrange equation method [31]. The vehicle vibration equations are established based on Lagrange equations and reorganized in matrix form as follows:

(19) $$\left[M_{V}\right]\left\{\ddot{Z}\right\}+\left[C_{V}\right]\left\{\dot{Z}\right\}+\left[K_{V}\right]\left\{Z\right\}=\left\{F_{Vb}\right\}$$

where $\left[M_{v}\right]$, $\left[C_{v}\right]$, and $\left[K_{v}\right]$ are the mass matrix, damping matrix, and stiffness matrix of the vehicle, respectively; $\left\{Z\right\}$, $\left\{\dot{Z}\right\}$, and $\left\{\ddot{Z}\right\}$ are the displacement, velocity, and acceleration vectors of the vehicle; $\left\{F_{Vb}\right\}$ is the force of the bridge on the vehicle.

A dynamic model of the bridge can be obtained through finite element modeling. The vibration equation of a bridge can be characterized as

(20) $$\left[M_{b}\right]\left\{\ddot{Y}\right\}+\left[C_{b}\right]\left\{\dot{Y}\right\}+\left[K_{b}\right]\left\{Y\right\}=\left\{F_{bv}\right\}-\left\{F_{g}\right\}$$

where $\left[M_{b}\right]$, $\left[C_{b}\right]$, and $\left[K_{b}\right]$ are the mass matrix, damping matrix, and stiffness matrix of the bridge; $\left\{Y\right\}$, $\left\{\dot{Y}\right\}$, and $\left\{\ddot{Y}\right\}$ are the displacement, velocity, and acceleration vectors of the bridge structure; $\left\{F_{bv}\right\}$ is the interaction force vector of the vehicle on the bridge; $\left\{F_{g}\right\}$ is the load vector of the wheels due to self-weight.

### 3.2. Road Surface Condition

Existing studies suggest that the RSC is one of the main factors affecting bridge vibration and DAFs [32,33]. It can be assumed to be a smooth stochastic process with zero mean for each state history and can be expressed by a power spectral density (PSD) function. In the current study, the multi-path RSC was generated using the inverse Fourier transform, and the following power spectral density (PSD) function [6] was used:

(21) $$G(n)=G\left(n_{0}\right){\left(\frac{n}{n_{0}}\right)}^{-2}\;$$

where G(n) denotes the roughness coefficient (m^3^/cycle); n is the spatial frequency; $n_{0}=0.1$ m^−1^. Three types of RSCs of ’good’, ’medium’, and ’poor’ represented by G(n) as per ISO-8608 (1995) [34] were considered; the roughness coefficients were $8\times 10^{-6}$ m^3^/cycle, $64\times 10^{-6}$ m^3^/cycle, and $128\times 10^{-6}$ m^3^/cycle for ’good’, ’medium’, and ’poor’ RSCs, respectively.

RSCs can be generated by a summation of a series of harmonics as given below:

(22) $$y(t)=\sum_{i}^{N}\sqrt{2G\left(n_{i}\right)\Delta n}\cos\left(2\pi n_{i}\beta t+\phi_{i}\right)$$

where $\phi_{i}$ is a random phase angle uniformly distributed from 0 to 2π; β represents a constant velocity; t represents a time corresponding to a particular location at which the roughness is to be found; N is the total number of terms used to build up the RSC sample in Equation (22). 

**Figure 2:**
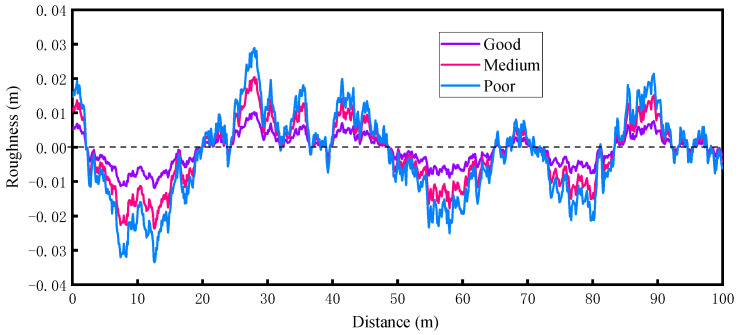
A sample of the road surface condition.

The randomness of the RSC interferes with the results of VBI analysis [35], and in this paper, 10 sets of samples were selected to eliminate its effect. A sample of the RSC is shown in Figure 2.

### 3.3. Vehicle-Bridge Interaction and Numerical Solution

**Figure 3:**
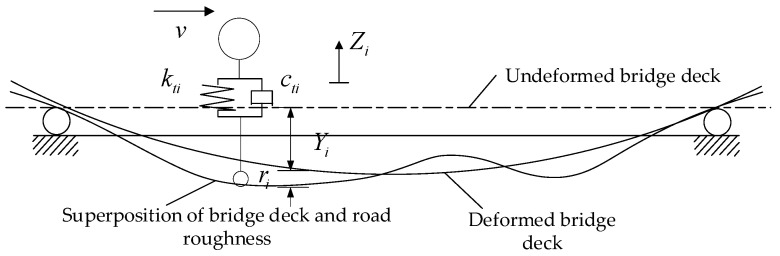
Displacement relationship between the wheel and bridge.

The vehicle’s ith tire is subjected to a force transmitted by the bridge as shown in Figure 3, and the relationship between the two is as follows:

(23) $$F_{vbi}=-k_{ti}\Delta_{i}-c_{ti}{\dot{\Delta }}_{i}$$

(24) $$\Delta_{i}=Z_{i}(t)-Y_{i}(x,t)-r_{i}(x)$$

(25) $${\dot{\Delta }}_{i}={\dot{Z}}_{i}(t)-{\dot{Y}}_{i}(x,t)-Y_{i}^{\prime }(x,t)v-r_{i}(x{)}^{\prime }v$$

where $F_{vbi}$ is the force of the bridge on the ith wheel; $k_{ti}$ is the stiffness of the ith wheel; $c_{ti}$ is the damping of the ith wheel; $\Delta_{i}$ is the spring compression of the ith wheel; $Z_{i}(t)$ and ${\dot{Z}}_{i}(t)$ are the vertical displacement and velocity of the ith wheel, respectively; $Y_{i}(x,t)$ and ${\dot{Y}}_{i}(x,t)$ are the vertical displacement and velocity of the bridge at the ith wheel; $r_{i}(x)$ is the road surface condition at the ith wheel; $Y_{i}(x,t{)}^{\prime }$ denotes the derivative of $Y_{i}(x,t)$ with respect to the longitudinal distance *x*; $r_{i}(x{)}^{\prime }$ denotes the derivative of $r_{i}(x)$ with respect to the longitudinal distance *x*.

The mode superposition method can be used to solve the dynamic equations of the bridge subsystem. When the mode superposition method is used, Equation (23) should be transformed into the following form:

(26) $$F_{\mathrm{vb}i}=-k_{ti}(Z_{i}-r_{i}-\langle N_{i}\rangle \Phi \left\{Q\right\})-c_{ti}({\dot{Z}}_{i}(t)-{\langle N_{i}\rangle }^{\prime }\Phi \left\{Q\right\}v-\langle N_{i}\rangle \Phi \left\{\dot{Q}\right\}-r_{i}^{\prime }v)$$

where $\langle N_{i}\rangle$ is the shape function of the element at the contact point between the wheel and the bridge deck; Φ denotes the vertical mode; $\left\{Q\right\}$ denotes the modal coordinate [30].

(27) $$F_{vbi}=-F_{bvi}$$

Assuming that the vehicle has *n* wheels, the force of the bridge on the vehicle can be expressed as follows:

(28) $$\left\{F_{Vb}\right\}=\left\{\begin{array}{l} \;0\\ \;\vdots \\ F_{vb1}\\ \;\vdots \\ \;0 \end{array}\right\}+\left\{\begin{array}{l} \;0\\ \;\vdots \\ F_{vb2}\\ \;\vdots \end{array}\right\}+\ldots +\left\{\begin{array}{l} \;0\\ \;\vdots \\ F_{vbn} \end{array}\right\}=\left\{\begin{array}{l} \;0\\ \;\vdots \\ F_{vb1}\\ \;\vdots \\ F_{vbn} \end{array}\right\}$$

After coordinate transformation, the final vibration equations of the vehicle-bridge coupled system are shown in the following:

(29) $$\left[\begin{array}{c} M_{b}\\ M_{v} \end{array}\right]\left\{\begin{array}{l} \ddot{Q}\\ \ddot{Z} \end{array}\right\}+\left[\begin{array}{cc} C_{b}+C_{b-b} & C_{b-v}\\ C_{v-b} & C_{v} \end{array}\right]\left\{\begin{array}{l} \dot{Q}\\ \dot{Z} \end{array}\right\}+\left[\begin{array}{cc} K_{b}+K_{b-b} & K_{b-v}\\ K_{v-b} & K_{v} \end{array}\right]\left\{\begin{array}{l} Q\\ Z \end{array}\right\}=\left\{\begin{array}{c} F_{b}\\ F_{v}+F_{G} \end{array}\right\}$$

where $C_{b-b}$, $C_{b-v}$, $C_{v-b}$, $K_{b-b}$, $K_{b-v}$, $K_{v-b}$, $F_{b}$, and $F_{v}$ are the vehicle-bridge interaction coupling parameters.

**Figure 4:**
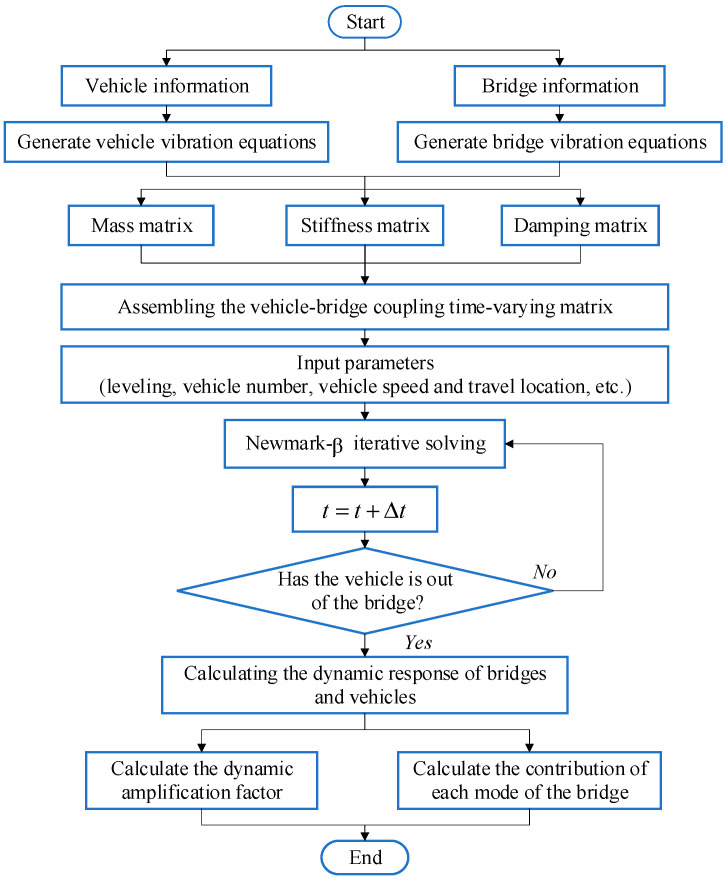
Flow of the vehicle-bridge coupling procedure.

**Figure 5:**
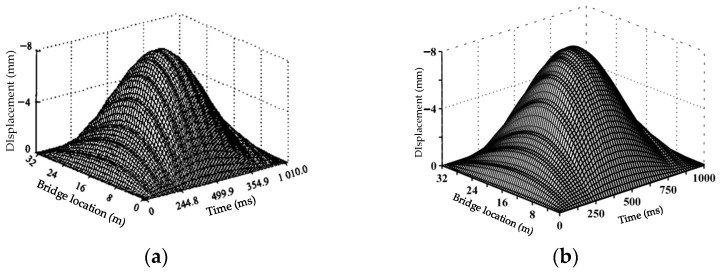
Comparison of the displacement responses at a speed of 40 km/h. (**a**) Reference [38]; (**b**) Self Programming.

The above vehicle-bridge coupled system may be solved using an uncoupled method [36,37]. Mode superposition makes it possible to separate the bridge modal analysis from the vehicle-bridge coupled model. In consequence, the number of calculations in Equation (29) and the complexity of the entire procedure are greatly reduced. The vibration equations of the vehicle-bridge coupled system are assembled automatically and solved in time history using the Newmark-β method. The program flow is shown in Figure 4. To verify the correctness of the vehicle-bridge coupling program, the vehicle parameters and bridge parameters from reference [38] were used; the calculation results of this paper’s program were compared and are shown in Figure 5. In the figure, the trend of the time course curve and the values of the mid-span displacement response are highly consistent with the reference [38]. The maximum value of the displacement response of the self-programmed program differs from the reference by 0.05 mm, with an error of only 0.6%, which fully demonstrates the accuracy of the vehicle-bridge coupling analysis method and the numerical calculation results in this paper.

## 4. Dynamic Analysis and Results

### 4.1. Prototype Bridges and Vehicle

**Figure 6:**
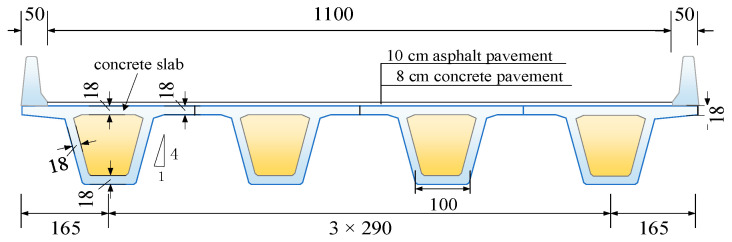
Typical cross-section of the bridge (unit: cm).

**Figure 7:**
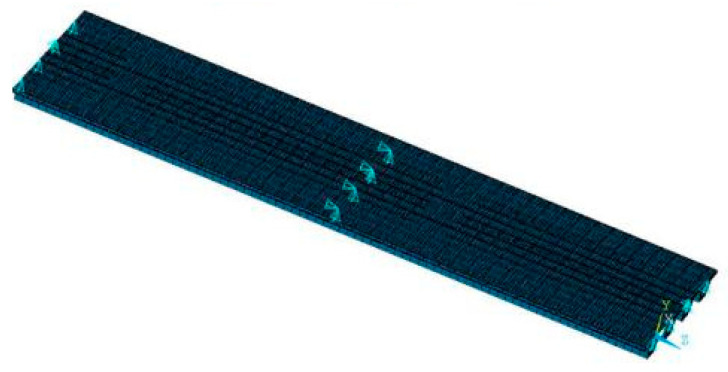
FEM of a 2 × 20 m continuous girder bridge.

A parametric investigation of precast continuous concrete box-girder bridges was conducted by considering the factors of span length, span number, road conditions, and vehicle speed. The levels of each influence factor analyzed by VBI are listed in Table 1. The corresponding beam heights for precast concrete continuous girder bridges with spans of 20 m, 30 m, and 40 m are 1.2 m, 1.8 m, and 2 m, respectively, and the other parameters are shown in Figure 6. The bridge was simplified to a grillage model [18,21,39] by ANSYS Software with cross-sectional properties assigned to each beam element. The roller support was modeled by releasing the horizontal movement. However, the hinged support was constrained from any horizontal movement. All supports were constrained in the vertical direction but allowed to rotate around the support line. Figure 7 shows a typical FEM applied for the dynamic analyses of the bridges. Three-node 3D beam elements, each with six degrees of freedom, were used to model all concrete beams. The modulus of elasticity $E_{c}$ of the concrete was 3.45 × 10^4^ MPa, the density ρ was 2500 kg/m³, and the structural damping ratio was taken as 0.05. The 4 cm of concrete pavement and 18 cm of slab concrete formed a cross-section assigned to the girder elements. The rest of the 4 cm of concrete pavement and 10 cm of asphalt pavement were assigned as a uniform mass to the girder elements.

The following assumptions were applied for the finite element modeling of the bridge: (1) the mass, stiffness, and damping characteristics of the bridge structure are uniformly distributed along the longitudinal direction; (2) the interaction between the bearing and the foundation is not taken into account; and (3) the torsional and distortional deformation of the beam are not taken into account.

**Figure 8:**
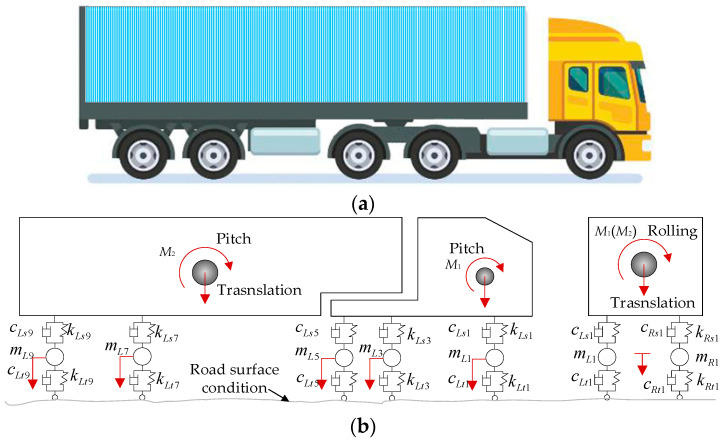
A 3D vehicle model with five axles: (**a**) prototype vehicle; (**b**) mechanical model.

The five-axle 3D vehicle with 15 degrees of freedom shown in Figure 8 was utilized for VBI analysis. Li et al. [40] stated the importance of 3D vehicle modeling in VBI simulations, which provides more realistic results compared to 2D models. The vehicle model consists of several rigid bodies connected by springs and dampers. More details of the vehicle can be found in [41].

As shown in Figure 8b, *M* is the mass of the vehicle body, *K* is the spring stiffness, *C* is the damping, *m* is the suspension mass, *L* represents the left-side wheel, *R* represents the right-side wheel, *s* represents the upper suspension, and *t* represents the lower tire.

**Figure 9:**
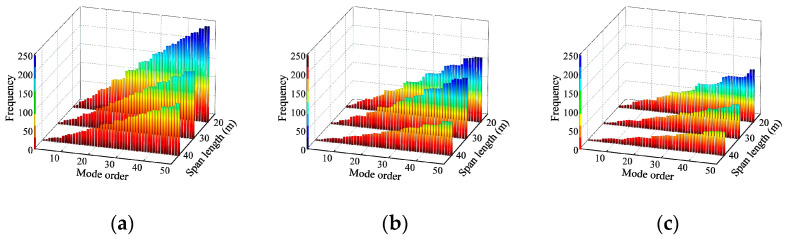
Bridge frequency: (**a**) two-span continuous girder bridges; (**b**) three-span continuous girder bridges; (**c**) four-span continuous girder bridges.

**Figure 10:**
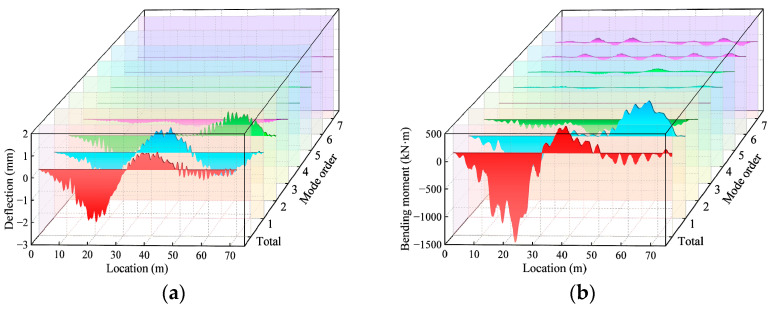
Decomposition of the total response: (**a**) deflection decomposition; (**b**) bending moment decomposition.

The first fifty vibration orders of modes and corresponding frequencies of the bridge were obtained using the Lanczos method as shown in Figure 9. The bridge frequencies become denser as the span number and length increase; bridges with more spans or a longer span require a higher-order mode to achieve the same level of frequency. Then, the mode superposition method was used to obtain the total dynamic response and the dynamic components of the bridge in each mode. Figure 10 shows the dynamic deflection decomposition process of the first seven orders of modes of a 3 × 20 m continuous girder bridge. The percentage of the effect associated with each mode out of the total effect is defined as the modal contribution $MPS_{k}$. The calculation process is shown in the following equation [29]:

(30) $$MPS_{k}=\frac{\sum_{j=1}^{n_{d}}\sqrt{\sum_{m=1}^{n_{t}}y_{k}^{j}{\left(t\right)}^{2}/n_{t}}}{\sum_{k=1}^{n_{m}}\sum_{j=1}^{n_{d}}\sqrt{\sum_{m=1}^{n_{t}}y_{k}^{j}{\left(t\right)}^{2}/n_{t}}}\;k=1\;to\;n_{m},j=1\;to\;n_{d}$$

where $y_{k}^{j}$ is the vertical modal displacement at structural node *j* for mode *k*; *n*_t_ is the total number of time steps; *n_m_* is the number of selected modes; *n*_d_ is the number of structural nodes; *k* is the time step.

### 4.2. Comparison of Dynamic Amplification Factors

(1) *Road surface condition*

The RSC is an important factor affecting the VBI and has been studied intensively [30,42,43]. This section investigates the effect of the RSC and bridge mode coupling on the DAFs of the deflection and bending moment. Four RSCs of ’smooth’, ’good’, ’medium’, and ’poor’ were used in the VBI analysis. The vehicle passed over the three continuous box-girder bridges (2 × 20 m, 3 × 20 m, and 4 × 20 m) at a constant speed of 80 km/h, and the dynamic responses (deflection and bending moment) of the bridges were obtained by considering different numbers of modes. Finally, the deflection DAF, bending moment DAF, and RDM were determined by Equations (15) to (16).

**Figure 11:**
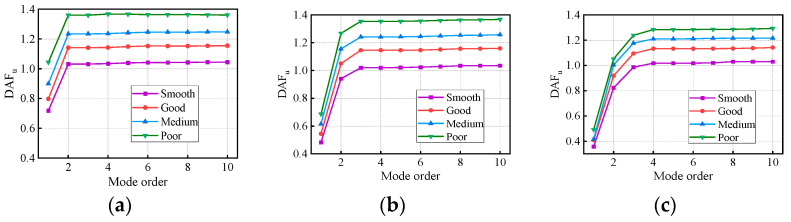
Deflection DAFs of the continuous girder bridges: (**a**) 2 × 20 m continuous girder bridge; (**b**) 3 × 20 m continuous girder bridge; (**c**) 4 × 20 m continuous girder bridge.

**Figure 12:**
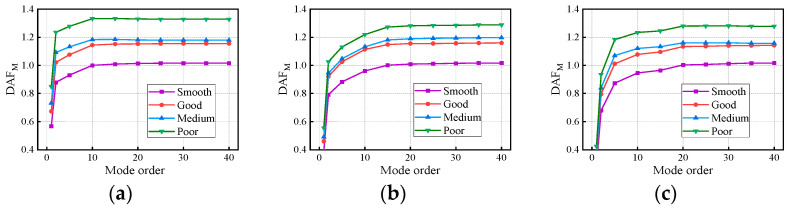
Bending moment DAFs of the continuous girder bridges: (**a**) 2 × 20 m continuous girder bridge; (**b**) 3 × 20 m continuous girder bridge; (**c**) 4 × 20 m continuous girder bridge.

**Figure 13:**
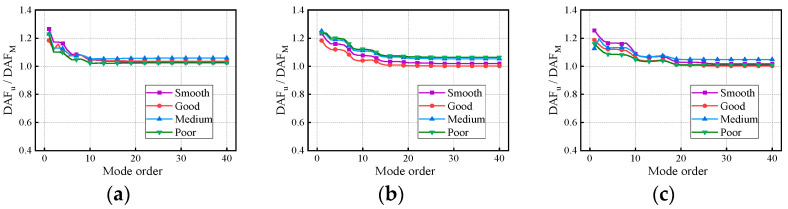
RDMs of the continuous girder bridges: (**a**) 2 × 20 m continuous girder bridge; (**b**) 3 × 20 m continuous girder bridge; (**c**) 4 × 20 m continuous girder bridge.

Figure 11 and Figure 12 present the variation in the DAFs of the deflection and bending moment with the modes and RSCs. The DAFs of the deflection and bending moment both increased with the RSC. For the three bridges, the maximum difference in the deflection DAF under the four RSCs is 0.32, while that in the bending moment DAF is 0.31. Therefore, periodic maintenance of the road surface can reduce the impact effect of vehicle loads on the bridge structure and improve vehicle occupants’ traveling comfort and safety on the bridge structure. In Figure 13, the RDMs of the three bridges all first decrease and then stabilize with the modes under the influence of RSCs. The convergent RDMs of the three bridges under the influence of RSCs range from 1.02 to 1.06, 1.00 to 1.06, and 1.00 to 1.05, respectively, indicating that the deflection DAF is greater than the bending moment DAF at the same location.

For the same RSC case, the deflection DAFs of the three bridges increase rapidly and then gradually stabilize with the vertical modes considered. The convergence mode (CM) is defined as the highest mode considered when the DAF first reaches stability. From Figure 11 and Figure 12, it can be seen that the maximum CM for the deflection DAF is 10, while that for the bending moment DAF is 40 for all cases. The average contribution of the first two modes to the deflection DAF is more than 95%, while their average contribution to the bending moment DAF is only 90%. When the modes considered were increased from 10 to 20, the bending moment DAFs and deflection DAFs were increased by 4.1% to 6.7% and 0.4% to 0.6%, respectively. It can be seen that the higher-order modes have a greater influence on the dynamic moments compared to the dynamic deflections for all continuous girder bridges. A small bending moment DAF may be obtained when modes are poorly considered. The differences in mode contributions to the DAFs of the deflection and bending moment in the same bridge under ‘smooth’, ‘good’, ‘medium’, and ‘poor’ RSCs are all within 5%. The above results show that the RSC does not significantly change the contribution of each mode in the dynamic response, or the CM, but it significantly affects the dynamic responses and DAFs.

(2) *Vehicle Speed*

**Figure 14:**
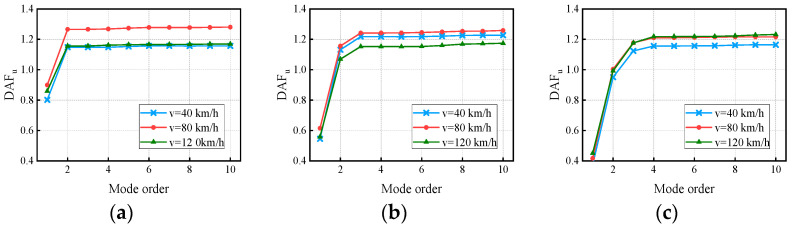
Deflection DAFs of the continuous girder bridges: (**a**) Results of the 2 × 20 m continuous girder bridge; (**b**) Results of the 3 × 20 m continuous girder bridge; (**c**) Results of the 4 × 20 m continuous girder bridge.

**Figure 15:**
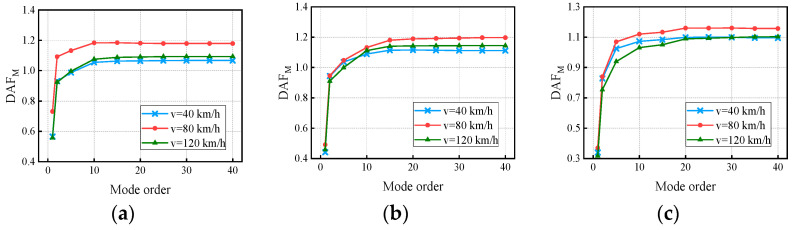
Bending moment DAFs of the continuous girder bridges: (**a**) Results of the 2 × 20 m continuous girder bridge; (**b**) Results of the 3 × 20 m continuous girder bridge; (**c**) Results of the 4 × 20 m continuous girder bridge.

**Figure 16:**
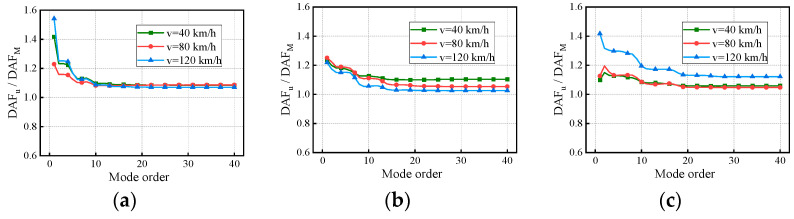
RDMs of the continuous girder bridges: (**a**) Results of the 2 × 20 m continuous girder bridge; (**b**) Results of the 3 × 20 m continuous girder bridge; (**c**) Results of the 4 × 20 m continuous girder bridge.

The effect of vehicle speed on DAFs has been demonstrated. Although many researchers have investigated the effect of vehicle speed on bridge response, this section focuses on the effect of vehicle speed on the CM and attempts to investigate the variation in the relationship between the deflection DAF and the bending moment DAF under the coupling of the vehicle speed and bridge modes. In the VBI analysis, the vehicle passed over the three bridges (2 × 20 m, 3 × 20 m, and 4 × 20 m continuous box-girder bridges) with a ’medium’ RSC at a constant speed of 40 km/h, 80 km/h, and 120 km/h. The DAFs obtained from the dynamic deflection and dynamic bending moment are shown in Figure 14 and Figure 15, respectively. Furthermore, the RDMs achieved by the combination of Figure 14 and Figure 15 are presented in Figure 16.

In Figure 15 and Figure 16, the convergent deflection DAFs, bending moment DAFs, and RDMs of the bridges do not increase or decrease with vehicle speed. The differences in the deflection DAFs after considering the CM for the three bridges under the influence of vehicle speed are 0.18, 0.2, and 0.13, respectively, while those of the bending moment are 0.12, 0.08, and 0.07, respectively. The convergent RDMs of the three bridges under the influence of the RSCs range from 1.07 to 1.09, 1.03 to 1.10, and 1.05 to 1.12, respectively, indicating that the deflection DAF is greater than the bending moment DAF at the same location. The mechanism of influence between the vehicle speed and DAF has been proven to be complicated, and most scholars have attempted to interpret that in regard to the critical speed [19,30,44]. However, this is not the focus of discussion in this paper, so the mode-related results are discussed further.

For the same vehicle speed, the deflection DAFs and bending moment DAFs of the three bridges increase rapidly and then gradually stabilize with the vertical modes considered. Conversely, the RDMs of the three bridges first decrease and then stabilize with the modes under the effect of vehicle speed. The maximum CM of the deflection DAF influenced by vehicle speed for the three bridges is 10, while that of the bending moment DAF is 40. The average contribution of the first two modes to the deflection DAF is more than 95%, while the average contribution to the bending moment DAF is only 88% for all cases. When the modes considered were increased from 10 to 20, the bending moment DAFs and deflection DAFs were increased by 4.1% to 6.7% and 0.4% to 0.6%, respectively. For all continuous girder bridges, the higher-order modes have a greater influence on the dynamic moments compared to the dynamic deflections even when influenced by the vehicle speed. The maximum difference in the contribution of the first-order modes for the deflection DAF induced by various vehicle speeds is 6%, while that for the bending moment DAF is 11%. The above results indicate that the vehicle speed affects the contribution of each mode in the DAF to some extent, which, in turn, alters the DAF. Through Equations (15) and (16), it can be concluded that v/l and $\beta_{n}$ are the major factors affecting the DAFs of the deflection and bending moment; vehicle speed exerts an impact on $\Omega_{n}$, consequently influencing $\beta_{n}$, ultimately resulting in alterations to the contribution of each mode.

(3) *Span Length*

**Figure 17:**
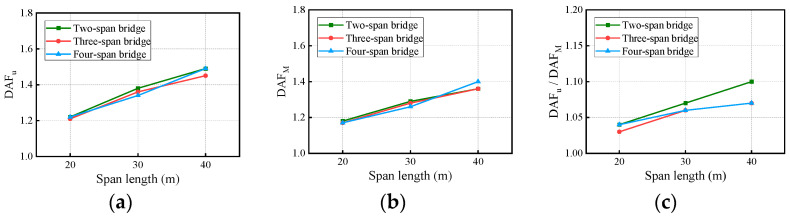
Bridge DAFs and RDMs influenced by span length: (**a**) deflection DAFs; (**b**) bending moment DAFs; (**c**) RDMs.

Many countries’ design codes have defined the DAF as solely a function of the span length, such as the U.S. (1992), China (1989), New Zealand (2013), Europe (2003), and Japan (1996) [19]. The span is a significant structural factor affecting the DAFs. In the analysis, the vehicle passed over nine continuous box-girder bridges (2 × 20 m, 2 × 30 m, 2 × 40 m, 3 × 20 m, 3 × 30 m, 3 × 40 m, 4 × 20 m, 4 × 30 m, and 4 × 40 m) with a ’medium’ RSC at a constant speed of 80 km/h. Bridge span changes can exacerbate the randomness of road surface conditions. For each bridge, 10 RSC samples were considered for analysis, and the final averaged DAFs and RDMs are plotted in Figure 17.

In Figure 17, the deflection DAF, bending moment DAF, and RDM all increase with the span length. The bridge RDMs for all cases range from 1.03 to 1.10, indicating that the deflection DAF is greater than the bending moment DAF.

**Figure 18:**
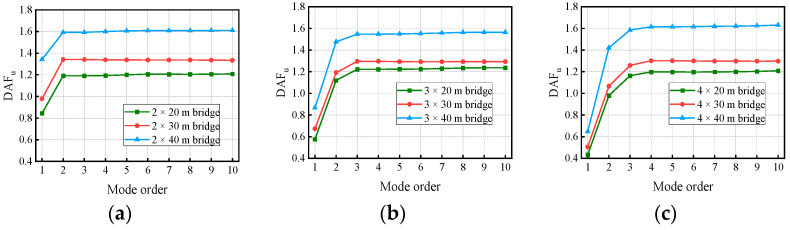
Deflection DAFs of the bridges: (**a**) DAFs for two-span continuous girder bridges; (**b**) DAFs for three-span continuous girder bridges; (**c**) DAFs for four-span continuous girder bridges.

**Figure 19:**
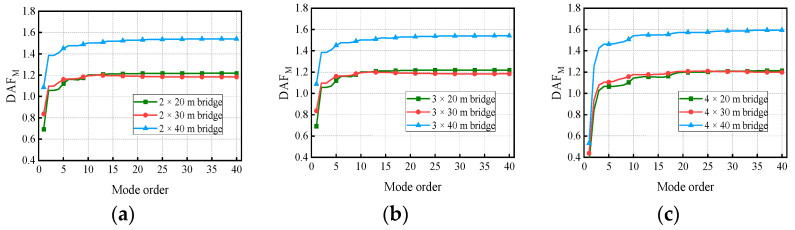
Bending moment DAFs of the bridges: (**a**) DAFs for two-span continuous girder bridges; (**b**) DAFs for three-span continuous girder bridges; (**c**) DAFs for four-span continuous girder bridges.

**Figure 20:**
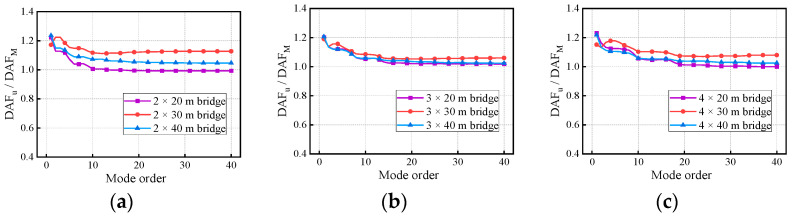
RDMs of the bridges: (**a**) RDMs for two-span continuous girder bridges; (**b**) RDMs for three-span continuous girder bridges; (**c**) RDMs for four-span continuous girder bridges.

Figure 18 and Figure 19 show the variation in the deflection DAF and bending moment DAF with the mode for one of the samples, while Figure 20 shows the results for the corresponding RDMs. The deflection DAF and bending moment DAF of bridges with the same span numbers all converge gradually with the modes, while the bridge RDMs decrease with the mode. The effect of span length on the first-order modal contribution is the maximum at 13.9%, and its effect gradually decreases as the mode order increases. The maximum CM for the deflection DAF is 10, while that for the bending moment DAF is 40 for all cases; the average contribution of the first two modes to the deflection DAF is more than 80%, while the average contribution to the bending moment DAF is only 69.5%. When the modes considered were increased from 10 to 20, the bending moment DAFs and deflection DAFs were increased by 1.1% to 4.5% and 0.0% to 0.4%, respectively. This indicates that the higher-order modes have a greater influence on the bending moment compared to the dynamic deflection for all continuous girder bridges.

(4) *Span Number*

Differences in the support conditions or span number could lead to differences in the response in the bridge even when subjected to the same loads. The data for bridges with the same spans in Figure 17, Figure 18, Figure 19 and Figure 20 were further summarized to examine the interactive effects of span number and mode on bridge DAFs and the RDMs.

**Figure 21:**
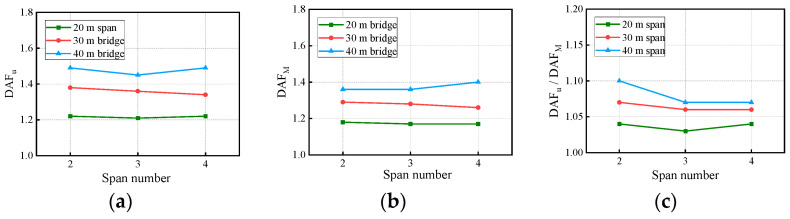
Bridge DAFs and RDMs influenced by span number: (**a**) deflection DAFs; (**b**) bending moment DAFs; (**c**) RDMs.

In Figure 21, the deflection DAF, bending moment DAF, and RDM show no clear relationship with the span number. The bridge deflection DAFs are all larger than the bending moment DAFs under the influence of the span number.

**Figure 22:**
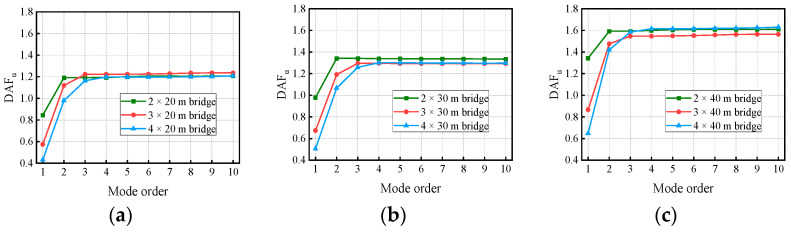
Deflection DAFs of the bridges: (**a**) DAFs for continuous girder bridges with 20 m spans; (**b**) DAFs for continuous girder bridges with 30 m spans; (**c**) DAFs for continuous girder bridges with 40 m spans.

**Figure 23:**
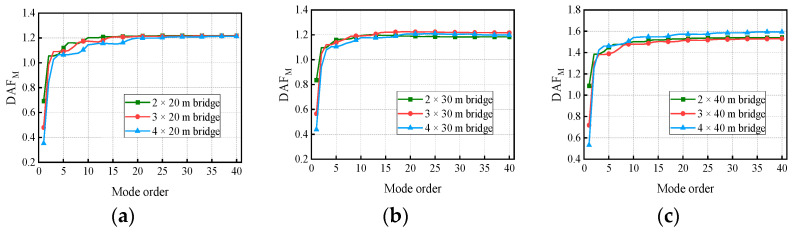
Bending moment DAFs of the bridges: (**a**) DAFs for continuous girder bridges with 20 m spans; (**b**) DAFs for continuous girder bridges with 30 m spans; (**c**) DAFs for continuous girder bridges with 40 m spans.

Figure 22 and Figure 23 show the variation in the deflection DAF and bending moment DAF with the mode and span number for one of the samples. For the deflection DAF, two-span bridges, three-span bridges, and four-span bridges increase rapidly in the first two, first three, and first four orders of modes, respectively. In contrast, the bending moment DAFs of the bridges with two, three, and four spans increase rapidly in the first 10, 15, and 20 orders of mode, respectively, and then gradually stabilize. The CM of the deflection DAF gradually increases with the span number, e.g., the CMs of two-span, three-span, and four-span continuous girder bridges are of the 5th, 8th, and 10th orders, respectively. Meanwhile, for continuous girder bridges of four spans or less, the necessary CM of the bending moment DAF is 40.

**Figure 24:**
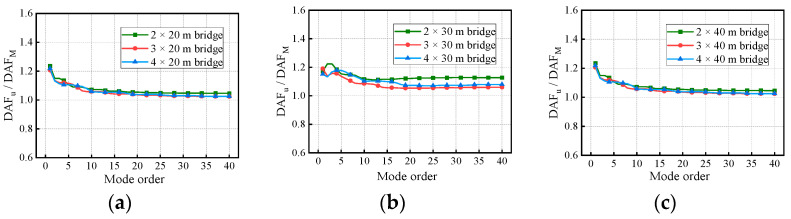
RDMs of the bridges: (**a**) RDMs for continuous girder bridges with 20 m spans; (**b**) RDMs for continuous girder bridges with 30 m spans; (**c**) RDMs for continuous girder bridges with 40 m spans.

In addition, there is a 43.7% difference in the contribution of the first-order mode to the deflection DAF for the two-span bridge compared to the four-span bridge. The effect of span number on the other order modal contributions gradually decreases as the mode order increases. A phenomenon similar to the above was found for the bending moment DAF. The various spectral characteristics of the bridges are the major reason for the differences in the above conclusions. The contribution of the first three modes to the bending moment DAF is much smaller compared to that for the deflection DAF in continuous girder bridges, indicating that the bending moment DAF is influenced by higher-order modes. The RDM results in Figure 24, which are affected by the span number, have been discussed in terms of the span length factor.

## 5. Cut-Off Modes for the Deflection and Bending Moment

Four RSCs of ‘smooth’, ‘good’, ‘medium’, and ‘poor’, and three speeds of 40 km/h, 80 km/h, and 120 km/h were considered in the VBI analysis. Each bridge contains twelve cases. A total of 216 dynamic responses containing the deflections and bending moments of the nine precast continuous concrete box-girder bridges were obtained. After statistical analysis for these bridges, it was found that the RDMs were in the range of 1.00 to 1.12, and the deflection DAFs were always greater than the bending moment (strain) result. It is possible to obtain safer DAFs by adopting the deflection instead of the bending moment (strain) in the evaluation of a bridge’s dynamic characteristics or the development of DAF criteria.

**Figure 25:**
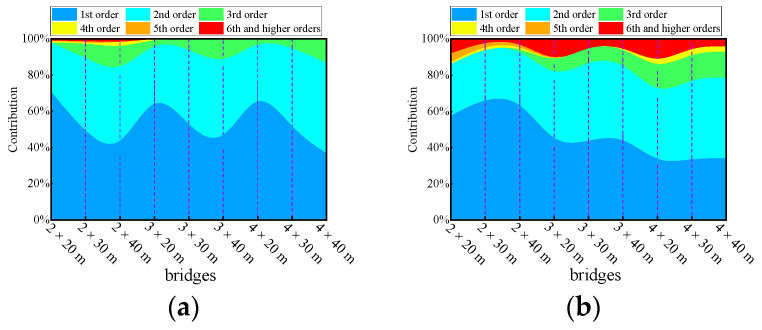
Mode participation for the continuous girder bridges: (**a**) mode participation for deflection; (**b**) mode participation for the bending moment.

The contribution of each mode for each bridge was averaged over the twelve cases and visualized in Figure 25. It can be observed that the contribution of each mode to the deflection DAF or bending moment DAF varies depending on the type of bridge, and the span number also influences the dominant vibration modes. The cumulative contribution of modes to the deflection response for two-span, three-span, and four-span bridges can exceed 95% when considering the first two, first three, and first four orders of modes, respectively. By considering modes beyond the 10th order, all modal dynamic effects can be accounted for, enabling accurate results for the deflection DAF. It is worth noting that for an equivalent level of cumulative modal contribution, bending moments consistently require a greater number of modes.

To summarize, the dominant vibration modes of the deflection and bending moment for the mid-span section of a continuous girder bridge are of the first three orders. Currently, many national code provisions utilize the fundamental frequency to calculate DAFs [44,45,46]. However, when determining the deflection DAF or bending moment DAF for continuous girder bridges, it is crucial to consider the impact of modal contributions from the second and third orders, in addition to the fundamental frequency. In addition, two types of accuracy indexes are proposed in combination with practical engineering application requirements. The first type of accuracy index, P_1_, is defined as a modal contribution of 95% and has a required minimum number of modes known as the cut-off mode K_1_. The second type of accuracy index, P_2_, is based on a modal contribution of 99% and has a cut-off mode of K_2_. The first type of accuracy index is used for data filtering and noise elimination in field measurements without distortion. The second type of accuracy index is applied in VBI analysis to enhance solution efficiency. 

After the statistical analysis, Table 2 provides the required cut-off modes for the accuracy indexes of the different bridge responses. The first type of accuracy index could be applied for data filtering and noise elimination in field measurements without distortion. It is worth noting that the cut-off mode can only eliminate high-frequency noise signals, while noise signals below the cut-off mode cannot be processed. The second type of accuracy index is utilized in VBI analysis to improve the efficiency of the solution. The mode superposition method, which does not require validation, is characterized by increased efficiency in VBI analysis when fewer modes are considered. Additional experimentation is required to validate the usefulness and effectiveness of the cut-off frequency.

## 6. Experimental Investigation

**Figure 26:**
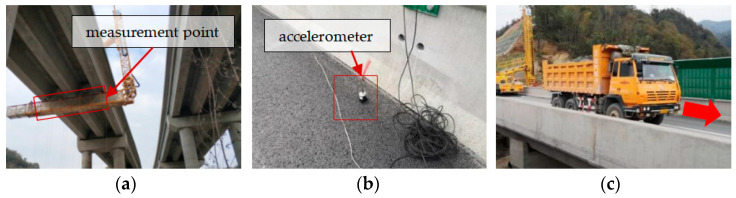
Field photographs: (**a**) deflection and strain sensor installation; (**b**) accelerometer arrangement; (**c**) dynamic test.

Two prestressed concrete continuous box-girder bridges, measuring 4 × 30 m and 4 × 20 m, were chosen for dynamic load tests on the Anlan Expressway in Shaanxi Province, China. The width of both bridges is 8.5 m. Details of the bridge sections can be found in the *Chinese General Bridge Atlas*. Strain measurements were performed using resistive, full-bridge circuit strain gauges with a double cantilever configuration. The measuring gauge length was 80 mm. These sensors have the advantages of a stable measurement value, high precision, high sensitivity, strong anti-interference ability, self-temperature compensation, wide adaptability, and easy installation and disassembly. Dial gauges with a resolution of 0.01 mm were used for deflection measurement. For the first bridge, strain gauges were applied to the bottom plate of the center girder at the mid-span of the side span, while the second bridge had strain gauges placed on the secondary side span. A visual inspection of the concrete in the bottom slab of the main girder was carried out before the measurement, and no apparent cracks were found. Suspension hammer systems including dial gauges were additionally installed near the strain gauges to measure the dynamic deflection. Furthermore, a piezoelectric accelerometer was attached to the bridge deck in the vicinity of the mid-span section; the sensitivity was 200 mV/g. A 36-ton truck with three axles passed over the bridge at a constant speed along the center of the bridge. Data were recorded using DASYLAB software. The sampling frequency was 200 Hz, and the measurement details are shown in Figure 26.

**Figure 27:**
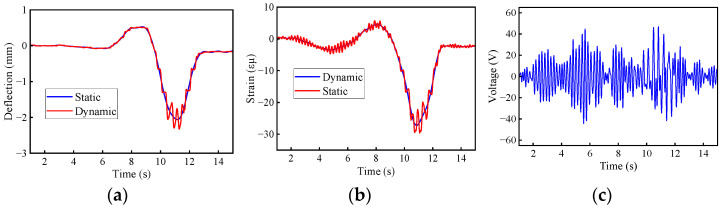
Measured dynamic responses for the 4 × 30 m bridge: (**a**) deflection; (**b**) bending moment; (**c**) acceleration.

**Figure 28:**
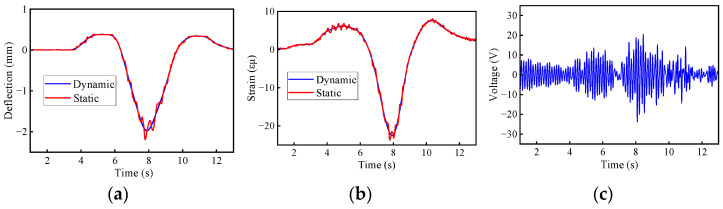
Measured dynamic responses for the 4 × 20 m bridges: (**a**) deflection; (**b**) bending moment; (**c**) acceleration.

The static deflection and static bending moment were obtained by applying low-pass filtering [47,48]. In Figure 27 and Figure 28, the calculated deflection DAFs for the two bridges were 1.13 and 1.11, while the strain (bending moment) DAFs were 1.09 and 1.06, respectively. The RDMs for the two bridges were 1.04 and 1.05, respectively, indicating that the deflection DAF at the mid-span location of the bridges was greater than the strain (bending moment) results. Only the first three orders of modes were obtained from the acceleration signals and were used to modify the finite element model. The higher modes can be extracted from the modified FE model, such as cut-off modes. To filter out high-frequency noise, a low-pass filter with a cut-off mode of K_1_ was applied. For deflection, the K_1_ order modal frequencies of the two bridges are 5.73 Hz and 9.8 Hz, while the frequencies for the bending moments are 16.5 Hz and 27.8 Hz, respectively. The deflection DAFs of the two bridges increased by 0.1% and 0.3% after noise elimination, while the strain DAF remained unchanged. Although the effect of noise elimination is not apparent due to the presence of a small noise component in the measured signal, it does highlight the effectiveness of the cut-off modal frequency in preserving signal fidelity.

## 7. Conclusions

In this paper, the association of the deflection DAF, bending moment DAF, and RDM with modes under multi-factor influence was investigated by numerical analyses. Two types of DAF accuracy indexes were proposed for VBI analysis and signal noise elimination. Finally, the relationship between the deflection DAF and bending moment DAF was verified by an experimental study, and the utility of cut-off modes in signal processing was demonstrated. The following conclusions were obtained: The deflection DAF and bending moment DAF of a continuous beam are related to the cross-sectional position and static load factor and are affected by the frequency ratio, damping ratio, vehicle speed, and span length. The number of modes also affects the DAF accuracy; the more modes are considered, the closer the result is to the analytical solution.

(1) Under varying conditions of travel speed, RSC, span length, and span number, the deflection DAF and positive bending moment DAF of the mid-span section of the continuous girder bridge increased rapidly with the modes and then stabilized. Under the influence of the above factors, the RDM decreased gradually with the number of modes but was greater than 1, indicating that the deflection DAF is always greater than the bending moment DAF.
(2) The span length, RSC, vehicle speed, and span number have strong, strong, complicated, and weak associations with DAF, respectively. The RSC did not significantly change the contribution of each mode to the DAF and CM, but it had a significant effect on the bridges’ dynamic response and DAF. The vehicle speed altered the dynamic response, the DAF, and the contribution of each mode to the response. The span length had a greater effect on the modal contribution than did the span number.
(3) The dominant vibration modes of continuous girder bridges were of the first two to four orders, and it is unreasonable to calculate the bridge deflection DAF and bending moment DAF by considering only the fundamental frequency. For precast continuous concrete box-girder bridges with four spans or less, the deflection DAF and bending moment DAF could be obtained with 95% accuracy after considering the first four orders and the first nine orders of modes, respectively.
(4) In the field experiment, the RDMs of the mid-span section for the 4 × 30 m and 4 × 20 m continuous bridges were 1.04 and 1.05, respectively, demonstrating that the deflection DAF of the mid-span section of the bridges was greater than the strain (bending moment) results. After filtering at the cut-off mode frequency corresponding to the 95% accuracy index was considered, the maximum variation of the deflection DAF and bending moment DAF was 0.3%, proving the effectiveness of the cut-off mode in preserving signal fidelity.
(5) In this paper, box-girder bridges were investigated. For precast bridges with other cross-sections (T-beam or hollow slab), the above conclusions are also applicable when the modes exhibit similar characteristics. Bridges with various structural types (skewed or curved) need further research. A comparative analysis was conducted on various parameters under the dynamic load test conditions of a single vehicle on the bridge. The vehicle number and driving position may have a greater influence on the results of the cut-off frequency compared to the bridge deck width, girder section, and girder number and, therefore, also need to be further investigated.

## Figures and Tables

**Table 1:** Bridge and vehicle information.

Bridge Type	Span Length (m)	Span Number	Road Surface Condition	Vehicle Speed (km/h) (km/h)
Precast concrete continuous box-girder bridge	20	2	Smooth	40
	30	3	Good	80
	40	4	Medium	120
	—	—	Poor	—

**Table 2:** Cut-off mode orders for the DAFs of the deflection and bending moment.

Cut-Off Mode Order	Deflection			Bending Moment		
	Two-Span	Three-Span	Four-Span	Two-Span	Three-Span	Four-Span
*K*_1_	3	3	4	7	8	9
*K*_2_	6	7	8	15	20	25

## Data Availability

Data are contained within the article.
